# Predicting Space Radiation Single Ion Exposure in Rodents: A Machine Learning Approach

**DOI:** 10.3389/fnsys.2021.715433

**Published:** 2021-10-15

**Authors:** Matthew T. Prelich, Mona Matar, Suleyman A. Gokoglu, Christopher A. Gallo, Alexander Schepelmann, Asad K. Iqbal, Beth E. Lewandowski, Richard A. Britten, R. K. Prabhu, Jerry G. Myers

**Affiliations:** ^1^NASA Glenn Research Center, Cleveland, OH, United States; ^2^ZIN Technologies, Inc., Cleveland, OH, United States; ^3^Department of Radiation Oncology, Eastern Virginia Medical School, Norfolk, VA, United States; ^4^Universities Space Research Association, Cleveland, OH, United States

**Keywords:** space radiation, radiation research, rodent studies, cognitive impairment, machine learning, support vector machine, Gaussian naive Bayes, imbalanced datasets

## Abstract

This study presents a data-driven machine learning approach to predict individual Galactic Cosmic Radiation (GCR) ion exposure for ^4^He, ^16^O, ^28^Si, ^48^Ti, or ^56^Fe up to 150 mGy, based on Attentional Set-shifting (ATSET) experimental tests. The ATSET assay consists of a series of cognitive performance tasks on irradiated male Wistar rats. The GCR ion doses represent the expected cumulative radiation astronauts may receive during a Mars mission on an individual ion basis. The primary objective is to synthesize and assess predictive models on a per-subject level through Machine Learning (ML) classifiers. The raw cognitive performance data from individual rodent subjects are used as features to train the models and to explore the capabilities of three different ML techniques for elucidating a range of correlations between received radiation on rodents and their performance outcomes. The analysis employs scores of selected input features and different normalization approaches which yield varying degrees of model performance. The current study shows that support vector machine, Gaussian naive Bayes, and random forest models are capable of predicting individual ion exposure using ATSET scores where corresponding Matthews correlation coefficients and F_1_ scores reflect model performance exceeding random chance. The study suggests a decremental effect on cognitive performance in rodents due to ≤150 mGy of single ion exposure, inasmuch as the models can discriminate between 0 mGy and any exposure level in the performance score feature space. A number of observations about the utility and limitations in specific normalization routines and evaluation scores are examined as well as best practices for ML with imbalanced datasets observed.

## Introduction

Galactic Cosmic Radiation (GCR) is an inherent risk for crewed missions traveling beyond the magnetosphere encircling Earth (Pietsch et al., 2011; Delp et al., 2016). GCR originates from outside the solar system and is primarily composed of high-energy atomic nuclei (Longair, 1992). The effects that GCR has on human cognitive health performance remain an essential inquiry for deep space travel given the inevitable exposure of astronauts during long-duration flights. An astronaut on a planned mission to Mars will be exposed to an estimated 130 mGy of GCR per year (Slaba et al., 2016). To quantify the potential change in human cognitive abilities to these levels of GCR ions, rodent-based human surrogate models are commonly used (Chancellor et al., 2018). Such studies show that the aggregate exposure to less than 250 mGy of various ions could have concomitant effects on cognitive performance for rodents (Parihar et al., 2015; Kiffer et al., 2019), implying potential complications for humans in space mission success.

Testing rodents’ recognition memory, spatial memory, anxiety, and attention provides quantification of difference in health and performance between control and irradiated groups (Cekanaviciute et al., 2018). One such test, the Novel Object Recognition (NOR) test (Antunes and Biala, 2012; Lueptow, 2017), evaluates a rodent’s object recognition memory. Exposure to whole-body and head-only High atomic number (Z) and Energy (HZE) ions and protons is shown to cause impairment in NOR for mice (Haley et al., 2013; Poulose et al., 2017). Reports of NOR tests performed after exposure to GCR component ions show 300mGy ^48^Ti particle irradiated mice having significantly reduced recognition memory and 50 mGy irradiated ^48^Ti and ^16^O and 300mGy ^16^O having no reduced recognition memory when compared with the control rodents (Parihar et al., 2016). A similar object recognition study using exposure of ^56^Fe particle doses greater than those experienced by astronauts in long term missions shows a dose dependent impairment of irradiated rats in differentiating between novel and familiar objects in object recognition memory tasks (Rabin et al., 2009).

Executive functions are a crucial component for adaptive functioning and include aspects of cognitive flexibility, planning, conceptual reasoning, attention, and set shifting (Spinella, 2005). One rigorous assay for assessing executive function is the Attentional Set-shifting (ATSET) test (Birrell and Brown, 2000; Garner et al., 2006). The ATSET is a constrained cognitive flexibility test where a rodent is evaluated on its ability to discriminate between cues across varying perceptual modalities in order to retrieve a food reward. This test consists of seven stages where rats forage for a food reward placed inside a bowl associated with a digging media and/or scent which varies by stage (Birrell and Brown, 2000). The ATSET attempts to quantify the cognitive performance and ability of a rodent to adapt to changes in an environment through a series of stages that each requires utilization of specific regions of the brain (Heisler et al., 2015). Five cognitive processes are interrogated during the ATSET: Simple Discrimination (SD), Compound Discrimination (CD), Intra-Dimensional Shifting (IDS), Extra-Dimensional Shifting (EDS), and reversal learning. In reversal learning, a rodent first learns a discrimination rule and then, after demonstrating this learned behavior, must unlearn and reverse its choice (e.g., an unrewarded cue is now rewarded). This aims to assess cognitive flexibility in the subject. Stages of the ATSET involving reversal learning include the CD reversal (CDR), ID reversal (IDR) and ED reversal (EDR) stages. The medial prefrontal cortex regulates performance in SD (Bissonette et al., 2008) while the perirhinal cortical region regulates performance in the CD tasks (Norman and Eacott, 2004). Cognitive flexibility is evaluated in the reversal and extra-dimensional shift stages of the assay (Heisler et al., 2015). An attentional set forms when a subject learns rules that allow it to differentiate relevant from irrelevant cues with regards to various stimuli. A rodent’s ability to adapt to new conditions and rules are evaluated during these stages—analogous to an astronaut adapting to changes in the environment.

A 2014 investigation (Britten et al., 2014) observes that exposure from 150 or 200 mGy of 1 GeV/nucleon ^56^Fe particle irradiation inhibits ATSET performance in rats at all stages. The study further reports that performance decrement varies across tasks as the SD, CD, CDR, and Intra-Dimensional Shifting (IDR) ATSET tasks exhibit strong impairment at 200 mGy, some impairment at 150 mGy in the SD and CD tasks, and no significant impairment at 100 mGy in any of the tasks resulting from exposure to 1 GeV/nucleon ^56^Fe particle irradiation, implying threshold radiation levels may exist above which impairment exacerbates or deemed beyond acceptable degradation. The authors also observe performance decrements in male Wistar rats after exposure to 1 GeV/n doses in the CD stage when assessed at the 12 week post-irradiation time point, but no impairment at this same post-irradiation time in the SD and CDR stages. In a subsequent study (Jewell et al., 2018), the cognitive performance of rodents is evaluated post-exposure to single beam ^56^Fe ions with doses varying between 100 and 150 mGy. This study finds that exposure to even 100 mGy ^56^Fe impairs cognitive performance in the ATSET and that CD is impaired across all doses. In contrast, performance in other stages is impaired at only certain radiation doses. This study further illustrates that performance impairment resulting from given GCR exposure is not uniform across stages. One of the distinctions in this study is the prescreening stage, which aids in differentiating between rodents who suffer memory or performance impairment and those who have an inherent difficulty with the tasks at hand. Another such study (Britten et al., 2018) using prescreening of the male Wistar rats and 600 MeV/n ^28^Si particles illustrates the ability of rats to perform ATSET 12 weeks after exposure to 50 – 200 mGy of 600 MeV/n ^28^Si particles. The findings show that exposed rodents exhibit a uniform impairment in the SD task along with impairment in other tasks varying according to dose. A male Wistar rat investigation (Parihar et al., 2016) shows significant performance decrement in the CD stage at the 12-week post-irradiation point after low dose 50 mGy 1 GeV/n ^48^Ti particle irradiation. This signals an impairment in the subjects in identifying and concentrating on task relevant perceptual cues. Notably, no impairment is found for the SD and CDR stages at this 12-week post-irradiation point. Another 1 GeV/n ^48^Ti investigation (Hadley et al., 2016) on male Wistar rats subjected to doses from 100 to 200 mGy shows long-term cognitive performance impairment in specific ATSET stages when evaluated at 3 months post exposure. The findings demonstrate significantly impaired CDR performance at all doses, especially 200 mGy, and impaired CD performance at 100 and 150 mGy. Unlike other studies (Jewell et al., 2018) which show impaired SD with 1 GeV/nucleon ^56^Fe particle irradiation at 150 and 200 mGy, no significant impairment in SD ability is established from 1 GeV/n ^48^Ti irradiation. All of these investigations help substantiate the notion that HZE particle irradiation can induce attentional set-shifting impairment.

Studies generally evaluate performance measures holistically, such as with Mann—Whitney (Britten et al., 2014; Parihar et al., 2016), one-way analysis of variance (Parihar et al., 2016), and cohort analyses (Britten et al., 2021), but do not attempt unique ML classification of individuals. Traditionally, space radiation studies look at average cohort values of the relevant performance metric and see if they differ significantly between the sham, i.e., non-irradiated, and irradiated subjects (Britten et al., 2021). One drawback to this methodology is that the entire subject population usually does not undergo constant performance decrement. Another drawback is that these cohort analyses do not indicate the extent to which the performance of individual subjects differs from the non-irradiated subset (Britten et al., 2021). Recent studies illustrate the use of ML and ensemble methods to automate sleep scoring in rodents using electroencephalogram and electromyogram combination recordings (Gao et al., 2016; Exarchos et al., 2020). More broadly, ML enables the use of human subject-level data in a wide range of medical applications (e.g., bone physiology (Schepelmann et al., 2019), bioelectromagnetics (Halgamuge, 2020), clinical decision making (Chen et al., 2019) — including radiotherapy (Valdes et al., 2017)). Other applications (Cacao et al., 2018) use combinations of stochastic (including Monte-Carlo methods) and physics-based models to predict neuronal dendritic damages caused by exposure to low linear energy transfer radiation (e.g., X-rays, γ-rays and high-energy protons). In contrast, data-driven approaches make inferences directly from the data without the requisite understanding of the underlying physical mechanisms and may offer unique insights given their limited reliance on presumptions and potential prediction capabilities.

This paper explores the feasibility of using ML techniques to predict received radiation exposure on a rodent subject from their corresponding ATSET cognitive performance scores. The remainder of the paper is structured as follows. First, the ATSET experiments upon which the analysis is based are explained and discussed, including details about the dataset and normalization options. We then inspect the rationale behind using three classification ML algorithms with varying underlying approaches and discuss essential concepts such as class imbalance and the cross-validation routine. Lastly, we present results from three ML classification algorithms with different underlying mathematical approaches and discuss the utility, findings, and limitations in their application to this type of analysis.

## Materials and Methods

### Analysis Workflow

**FIGURE 1:**
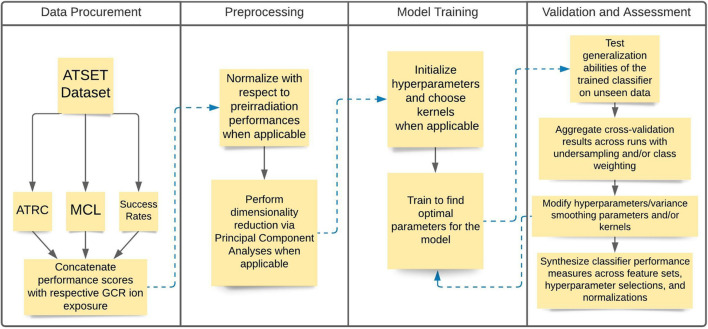
Flowchart diagram illustrating the general steps in the analysis methodology. The Data Procurement and Preprocessing stages consist of acquiring and segmenting the data and then performing the relevant normalizations and dimensionality reduction when applicable. The Model Training and Validation and Assessment stages consist of training the model using the relevant algorithms, evaluating their performance, and making relevant modifications to improve model training.

The analysis consists of a data acquisition phase where the experimental data are assembled, and a preprocessing stage where data are normalized and dimensionality reduction techniques are employed. Next, we initiate a model training phase where hyperparameters and other considerations of the model itself, such as kernels and class weighting, are specified. We conclude with a validation and assessment phase where cross-validation, hyperparameter tuning, and corresponding evaluation metrics are assessed. Figure 1 depicts a flowchart diagram of the general methodology in the analysis.

### Data Procurement: Irradiated Rodent Performance Data

We procure individualized rodent data for this numerical investigation from experimental ATSET results. The experimental data consist of previously reported 400 MeV/n ^4^He with LET of 1.2 keV/μm (Burket et al., 2021), 600 MeV/n ^28^Si with LET of 54 keV/μm (Britten et al., 2018), 1 GeV/n ^48^Ti with LET of 106 keV/μm (Hadley et al., 2016; Parihar et al., 2016), 600 MeV/n ^56^Fe with LET of 180 keV/μm (Jewell et al., 2018), and previously unreported 400 MeV/n ^16^O with LET of 19.4 keV/μm whole-body single beam ion exposures with a dose of 0 (sham), 10, 30, 50, 100, or 150 mGy on 10-month-old male Wistar rats evaluated before irradiation and at the 12 week post-irradiation time point. A tabulated summary of these studies is given in Table 1. These experiments are performed with multiple cohorts over a multi-year timespan in Dr. Richard Britten’s lab at East Virginia Medical School (EVMS; Norfolk, VA, United States). For the experimental tests from which performance decrement scores are calculated, the rodents perform the first four ATSET stages in the pre-irradiation phase, and all seven stages in the post-irradiation stage. The pre-irradiation results provide a baseline for some aspects of their innate performance abilities for a direct comparison to their post-irradiation performance and are a strong consideration in choosing to utilize the scores of this specific experiment.

**TABLE 1:** List of publications corresponding to the datasets used in this analysis.

Publication	Ion	Ion mass	Dose (mGy)	Linear energy transfer LET (keV/μm)	Kinetic energy (MeV/n)
Britten et al., 2014	Fe	56	100, 150, 200	150	1000
Hadley et al., 2016	Ti	48	100, 150, 200	106	1000
Parihar et al., 2016	Ti	48	50	106	1000
Britten et al., 2018	Si	28	50, 150, 200	54	600
Jewell et al., 2018	Fe	56	10, 30, 50, 100, 150	180	600
Britten et al., 2020	Si	28	10, 30, 50, 100, 150	54	600
Burket et al., 2021	He	4	10, 50, 100	1.2	400

*All studies use male Wistar rats, 10-months-old at time of irradiation. All irradiation is administered to the whole-body at the NASA Space Radiation Laboratory. All behavioral testing is performed in Dr. RB’s lab at the East Virginia Medical School at 12 ± 2 weeks post-irradiation. Irradiation details specific to each study are listed.*

To elucidate the ML approach to utilizing this individualized rodent data, a more comprehensive description of the ATSET test is beneficial. The attentional set-shifting test is an executive function task designed to quantify the cognitive performance of a rodent through the use of a food reward and a series of stages that use distinct centers of the brain when executed (Britten et al., 2014; Heisler et al., 2015). This multi-stage test investigates the ability of the rats to complete these seven stages with a performance evaluation using two different scores: the Attempts To Reach Criterion (ATRC) and the Mean Correct Latency time (MCL). The ATRC scores examine the number of attempts that the rat takes to pass a stage (the criterion to pass a stage is successful food retrieval in six consecutive attempts). Thus, a lower score in ATRC represents a higher-performing subject. Six is the lowest score possible, representing a perfect performance in that stage. The MCL metric evaluates the average time for successful retrieval of food; thus, higher magnitudes represent worse performance. We further define an additional score, the success rate, which provides the ratio of correct attempts to the sum of correct and wrong attempts. These three metrics, ATRC, MCL, and Success Rate, are henceforth also referred to as *evaluation scores*.

### Preprocessing Rodent Attentional Set-Shifting Data

We utilize specific data normalizations of input feature sets in predicting GCR ion exposure. Two types of normalizations of the data are performed to examine the rodent performance decrements relative to pre-irradiation performance: prescreen SD and 4-stage normalization. Prescreen SD normalization is the division of each of the post-irradiation performance scores in the SD, CD, CDR, IDS, IDR, EDS, and EDR stages by the corresponding prescreen values in the SD stage. 4-stage normalization focuses only on four post-irradiation scores, namely, SD, CD, CDR, and IDS, and divides each by its corresponding prescreen value. The prescreen values for the other, later stages of ATRC are not available for this type of normalization. Additionally, we utilize the raw post-irradiation data (i.e., non-normalized) to compare performance with the normalized datasets.

The adverse effects of high dimensional input feature spaces relative to the training set size require mitigation. This “curse of dimensionality” manifests when there are not enough samples to generalize well over the large input domain (Verleysen and François, 2005). Given the size for this dataset consisting of at most 62 individual rodents per dose, we utilize Principal Component Analysis (PCA) (Jolliffe and Cadima, 2016) to represent the training data in a reduced 2-D feature space from the 7-D (prescreen SD or non-normalized datasets) and 4-D (4-stage normalized datasets) feature spaces to alleviate the potential of higher dimension input challenges. PCA uses the input data to create a new orthonormal coordinate system, hosting a projection of the original features. The first axis of the new system is constructed in a way that maximizes the variance of the projected data, and the projected features on that axis are the first Principal Component (PC). The second axis holds the projection with the second to highest variance in the data, and so on. The choice of two dimensions in the PCA is due to the added benefit of visualization, but future approaches could seek to capture a certain desired percentage of the variance. PC transformed features are not deemed critical in this analysis considering that the number of input features varied throughout was not particularly high (4-D or 7-D in almost all cases), but subsequent performance after dimensionality reduction was deemed worthy of examination for insights as to whether or not dimensionality reduction is worthwhile.

### Classification Terminology

In keeping with standard ML terminology, different dimensionalities of the input variable feature space account for the number of features available. For the output, *binary classification* refers to the model application to predict one of two possible classes. In this study, we predict whether a rat is sham (dose 0 mGy) or irradiated with a particular dose or any dose of a certain ion. As an example, a “0 vs. 150” classification analysis attempts to make predictions on whether a given feature vector (vector of the scores in the ATSET stages as input to the ML model) belongs to the 0 mGy class or the 150 mGy class.

### Machine Learning Algorithms

Support vector machine (SVM) analysis (Noble, 2006) is a discriminative ML algorithm used for classification and regression tasks. SVM is considered a robust and effective classifier employed in medical data analysis research (Janardhanan et al., 2015). We summarize the application used in this analysis with respect to linear classifiers. In the SVM algorithm, the objective is to find a hyperplane that maximizes the margin between the closest data point of each class, the support vectors, and the hyperplane, called the decision boundary.

A linear classifier is expressed as a linear combination of the *d* features *x*_*i*_ of the input feature vector **x** as shown below:


(1) $$f\left(x\right)=\sum_{i=1}^{d}w_{i}x_{i}+b={\text{w}}^{T}\text{x}+b,$$

where **w** is a vector representing the weights or parameters of the linear classifier and b is a scalar representing the bias. A binary (two-class) classifier is trained to learn parameters that classify the output feature *y* as class 1 if *f* (***x***) ≥ 0 and class −1 if *f* (***x***) ≤ 0. Data, $D={\left\{x^{\left(j\right)},y^{\left(j\right)}\right\}}_{j=1}^{N}$, with *N* training examples is linearly separable if and only if:


$$\exists \text{w}\in {\mathbb{R}}^{n},b\in \mathbb{R}s.t.{\text{w}}^{T}{\text{x}}^{\left(j\right)}+b\geq 1\mathrm{if}y^{\left(j\right)}=+1,$$

(2) $${\text{w}}^{T}{\text{x}}^{\left(j\right)}+b\leq -1\mathrm{if}y^{\left(j\right)}=-1.$$

The width of the margin is given by $\frac{2}{{||w||}_{2}}$. Maximizing this margin is achieved by solving the quadratic optimization problem:


(3) $$min_{w,b}{\text{w}}^{T}\text{w},s.t.y^{\left(j\right)}\left({\text{w}}^{T}{\text{x}}^{\left(j\right)}+b\right)\geq 1\forall j\in \left\{1,\ldots ,N\right\}$$

The use of a kernel function obviates the need to compute the dot product ***w***^*T*^***x*** in the higher dimensional feature space by replacing this calculation and hence reducing the computational burden. Through a kernel function, one can construct decision boundaries that are linear in a higher dimensional feature space but nonlinear in the original feature space. Thus, nonlinear decision boundaries can be drawn in the original feature space to still perform under possible nonlinearities in the data distribution. For this investigation, we utilize the linear, radial basis, and third-degree polynomial kernel functions. Results are reported as averages across all the kernels unless stated otherwise. Slack variables can also be introduced to allow for a certain number of outliers to fall on the wrong side of the hyperplane when training (Smola and Schölkopf, 2004). This is important for applications where outliers are to be expected like in the medical sciences.

Gaussian naive Bayes (GNB), a probabilistic model which assumes that features are conditionally independent of each other (Mitchell, 1997), represents the second ML application. GNB relies on Bayes Theorem to find the class that the sample most likely belongs to. For a feature vector ***X*** = < *x*_1_, *x*_2_, *x*_*n*_ > and the *i*^*th*^ class label *C_i_*, Bayes Theorem is shown in eq. (4) where *P* (*C*_*i*_| ***X***) is the posterior probability, *P* (***X*** | *C*_*i*_) is the likelihood, *P*(*C*_*i*_) is the prior probability, and *P*(***X***) is the evidence/marginal likelihood.


(4) $$P\left(C_{i}|X\right)=\frac{P\left(X|C_{i}\right)P\left(C_{i}\right)}{P\left(X\right)}$$

The likelihood can be reformulated as the following using the probability chain rule (eq. 5).


$$P\left(X|C_{i}\right)=P\left(x_{1},x_{2},\ldots x_{n}|C_{i}\right)=P\left(x_{1}|x_{2},\ldots x_{n},C_{i}\right)\ldots$$

(5) $$P\left(x_{n-1}|x_{n},C_{i}\right)P\left(x_{n}|C_{i}\right)$$

In GNB, we assume the conditional independence of the *n*number of features, such that the likelihood, can be expressed as shown in eq. (6).


(6) $$P\left(X|C_{i}\right)=P\left(x_{1},x_{2},\ldots x_{n}|C_{i}\right)=\prod_{j=1}^{n}P\left(x_{j}|C_{i}\right)$$

The posterior probability can be written now by substituting the likelihood back into the model as seen in eq. (7).


(7) $$P\left(C_{i}|\text{X}\right)=\frac{\left[\prod_{j=1}^{n}P\left(x_{j}|C_{i}\right)\right]P\left(C_{i}\right)}{P\left(X\right)}$$

In GNB, we seek to find the most probable hypothesis. The Naive Bayes classifier is hence a function that assigns a class based on which class label obtains the highest value of the posterior.

The marginal likelihood in the denominator will not change given the input; thus, the optimization can be written as shown below.


(8) $$\begin{array}{c} \text{argmax}\\ i\in \left\{1,\ldots ,I\right\} \end{array}\;P\left(C_{i}\right)\prod_{j=1}^{n}P\left(x_{j}|C_{i}\right)$$

An assumption to be noted is that we use a uniform prior for the GNB model. This is because the number of samples for each dose is not assumed or expected to be representative of its prior probability.

**FIGURE 2:**
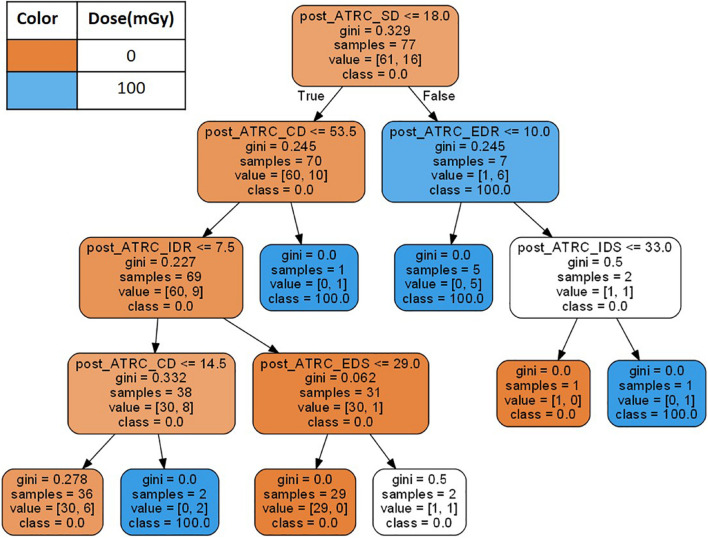
A decision tree example created using the 7 stage raw ATSET performance scores as features. The Gini coefficient is evaluated to determine which features using the ATSET scores best segregate the classes of sham rats (0 mGy) and those irradiated with 100 mGy of Fe.

The third ML approach incorporates the data within a decision tree (DT) analysis. Decision tree models (Breiman et al., 1984) represent ML approaches that predict values of a target output based on input variables (features) following hierarchical if-then-else decision rules and allocating samples to nodes as shown in Figure 2. Decision tree classifiers find the best features to split such that the resulting daughter nodes are as well segregated as possible. The algorithm starts by allocating all samples in question to the root node and asking if samples have a score above or below a threshold, then splitting them accordingly. The decision of which score to pick to determine the threshold comes from the evaluation of an impurity index such as the Gini impurity (Sundhari, 2011), indicating the probability of a randomly selected sample being misclassified. A Gini impurity of 0 indicates that all samples belong to the same class. After the Gini impurity is calculated for all features using the samples in the node, the feature with the lowest Gini is selected to split the data. The process proceeds through more layers of the tree until an appropriate stopping criterion specified by the user is satisfied (Breiman et al., 1984) such as the maximum depth of the tree which is set to six in this analysis. Figure 2 shows a learned decision tree when trained using the ATSET data.

An extension to decision trees is Random Forests (RF) classification (Breiman, 1999; Tsipouras et al., 2018). A RF is constructed from an ensemble of decision trees where each trains over random data samples with random sets of input features to predict the output variable. The RF receives a vote for the class from each tree and selects the most popular class as the class predicted by the RF. The motivation for RFs is that outputs from single trees are very sensitive to noise in the training data whereas aggregating them reduces overfitting and bias due to the variation between the included trees.

These three algorithms are chosen due to their varying underlying approaches in order to evaluate performance across many different perspectives. SVM, GNB, and RF rely on geometric, probabilistic, and tree-based approaches, respectively. Ensemble methods could be employed for future analyses to assess whether all models could be leveraged in tandem for prediction. For instance, to improve precision or recall scores, one could set a decision heuristic to predict a given class only in the case SVM, GNB, and RF were all in agreement.

### Example Analysis

**FIGURE 3:**
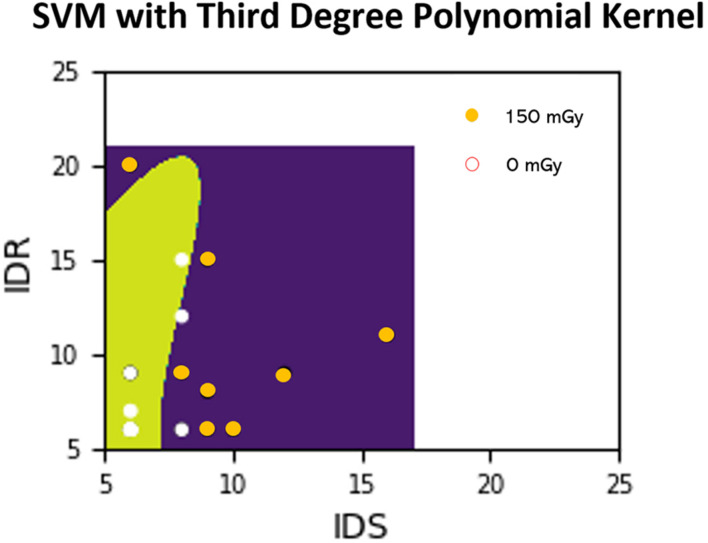
Example SVM analysis in a 2-D feature space where the ATSET performance scores IDS and IDR are used to predict whether a subject was irradiated with 150 mGy or from the control group (0 mGy). Points in the figure represent training points which we then perform predictions on after the model training. Areas shaded with green and purple are areas which the classifier predicts as coming from the non-irradiated subjects and the 150 mGy irradiated subjects, respectively. The decision boundary is delineated by the change from green to purple. A white point on the green shade and an orange point on the purple shade represent correct predictions.

For the objective of predicting exposure based on performance scores, ML algorithms described above such as SVM and GNB are implemented for training classifiers. These algorithms find the optimal parameters for a decision boundary that best separates the data into their respective classes (e.g., dose exposure). Once the model finds these parameters and the model is considered “trained,” then new data points can be tested to determine the most likely classification. Figure 3 depicts an example of the SVM classifier approach where the features (performance scores) are being used to predict the exposure dose (either 0 or 150 mGy in this case).

### Model Training and Validation

**FIGURE 4:**
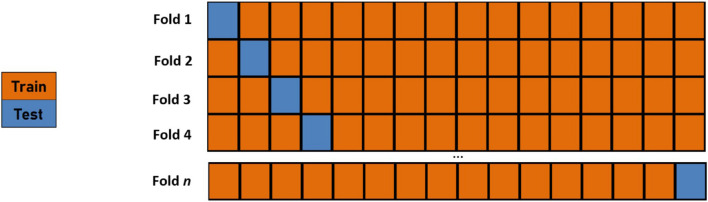
A graphical depiction of an *n*-fold leave-one-out cross-validation routine where *n* is the number of data points. The model’s parameters are trained using an *n-1* subset of the total number of examples and then tested using the instance left out in order to assess the generalization abilities of the learned classifier.

In ML analyses, to help ensure that correct classifications are not found via the wrong means, one should not only ensure that all the features used are realistic but also avoid using input features that carry information equivalent to what the output variables are predicting. In this analysis, only the cognitive performance scores are utilized as features which prevents said bias. Additionally, a robust cross-validation procedure should be performed to demonstrate a model’s generalization abilities. We employ a Leave-One-Out Cross-Validation (LOOCV) procedure (Efron, 1982) whereby the number of folds in the cross-validation routine is equal to the size of the training set (Figure 4). The model’s parameters are trained using an *n-1* subset of the total number of examples and then tested using the instance left out in order to assess the generalization abilities of the learned classifier. Thus, the training is performed for all the training examples, save one instance. This “left out” instance is subsequently tested using the learned parameters from the training, and the process is repeated over all training examples.

For reporting classification accuracies (eq. 9), an undersampling routine is utilized due to the inherent issues caused by certain dose classes that have much more data points than others (Chawla, 2005). A large class imbalance can become problematic when model learning begins to bias predictions towards the classes with a higher number of samples since high accuracy scores can often be achieved just by choosing the class with the larger class ratio. For instance, having decuple the number of training examples of a class 0 over class 1 may bias the model towards creating *de facto* majority vote classifiers. In this scenario, the model is not learning in its intended way, and raw accuracy scores obfuscate interpretation. To mitigate these effects when reporting accuracies, we employ an undersampling procedure as one of the class imbalance mitigation strategies. In this procedure, the classifier trains in a series of iterations by using the full training data of the class with the lowest number of training samples and a random subset of the training set for the class with the larger class ratio. In each training iteration, the number of training samples within each class label is equal, locally mitigating the obfuscation induced by class imbalance. The analysis is run over 100 iterations to try and reduce noise in the outputs and the average metrics reported over these iterations.

Another way of dealing with class imbalance is through class weighting, where weights are used to penalize incorrect classifications of the class with fewer samples, the minority class, more harshly than the class with more samples, the majority class. Undersampling is similar to class weighting by providing more weight to the minority classes since replicating certain observations during the model fitting stage increases the penalty for these observations as well. The results between the two differ due to the data splitting aspect. The results from using the methods of both class weighting and undersampling are presented and juxtaposed here. LOOCV is utilized in both cases.

For cross-algorithm comparisons and input feature set selection we use accuracy, Precision-Recall (PR) plots, F_1_ scores, and Matthews Correlation Coefficient (MCC) (Chicco and Jurman, 2020). These evaluation metrics are shown in eqs. 9-13. True Positive (TP), False Positive (FP) (or Type I error), True Negative (TN), and False Negative (FN) (or Type II error) refer to the ML outcome prediction vs. the actual class. The “true” or “false” signifier preceding the “positive” or “negative” denotes whether the classifier correctly (true) or incorrectly (false) predicted whether the sample was positive or negative. The True Positive Rate (TPR), also known as “sensitivity” or “recall,” is the number of true positives (occurs if the model predicts correctly when the test sample actually is positive) divided by the number of actual positives as seen in eq. (10). The precision is the number of true positives divided by the sum of the true positives and false positives, as shown in eq. (11). This is the ratio of the number of test samples that the model correctly predicts as positive over the total number of samples predicted positive in general (including the ones incorrectly classified as positive). PR curves are a means of assessing the diagnostic capabilities of classifiers. The PR curve often consists of a plot of the TPR on the *x*-axis vs. the precision on the *y*-axis, and the threshold probability for predicting is varied for each point in the curve. The best performing classifiers are towards the top right of the plots, signifying both high precision and recall. Precision and recall analysis is particularly adept at dealing with data with high-class imbalances (Davis and Goadrich, 2006).


(9) $$Accuracy=\frac{\left(TP+TN\right)}{\left(TP+TN+FP+FN\right)}$$

(10) $$Recall=\frac{TP}{\left(TP+FN\right)}$$

(11) $$Precision=\frac{TP}{\left(TP+FP\right)}$$

(12) $$F_{1}score\left(Fmeasure\right)=2*\frac{Precision*Recall}{Precision+Recall}$$

(13) $$MCC=\frac{\left(TP*TN\right)-\left(FP*FN\right)}{\sqrt{\left(TP+FP\right)\left(TN+FP\right)\left(TP+FN\right)\left(TN+FN\right)}}$$

Hyperparameters refer to modeler-defined parameters that are specified before training. Varying these hyperparameters across many different values is common (known as a grid search, Meng et al., 2013) in order to find the ones best suited for the analyses at hand. In this analysis, we show PR plots across hyperparameters instead of threshold probabilities (which one can think of as a hyperparameter of the decision rule rather than the model itself since the modeler post-training defines threshold probabilities) since we are not evaluating the tradeoff of type I and type II classification errors here. Hyperparameter tuning is performed for the SVM and GNB algorithms using their MCC results to determine the optimal hyperparameters for the model.

In assessing the performance of the classifiers, the corresponding F_1_ score and MCC are calculated as seen in eqs. (12) and (13). F_1_ scores are the harmonic mean of the precision and recall. MCC is a less commonly reported metric but often attested as being the prime binary classification evaluation metric for classification problems (Chicco and Jurman, 2020; Powers, 2020) since it takes into account true negative outcomes as well, unlike F_1_ scores. MCC represents a gauge of the linear correlation between two binary variables, in this case, the true class and predicted class (Chicco and Jurman, 2020; Powers, 2020).

When looking at ML performance classification results, it is important to make no preconceptions about what a “good” target evaluation measure (e.g., precision, recall, MCC, etc.) should be for a “good” classifier—as the economics proverb goes: “when a measure becomes a target, it ceases to be a good measure”—and instead evaluate performance based on circumstances and objectives. In other words, let the circumstances govern the thresholds of efficacy. This is the tradeoff with type I and type II classification errors.

## Results

### Data Visualization

**FIGURE 5:**
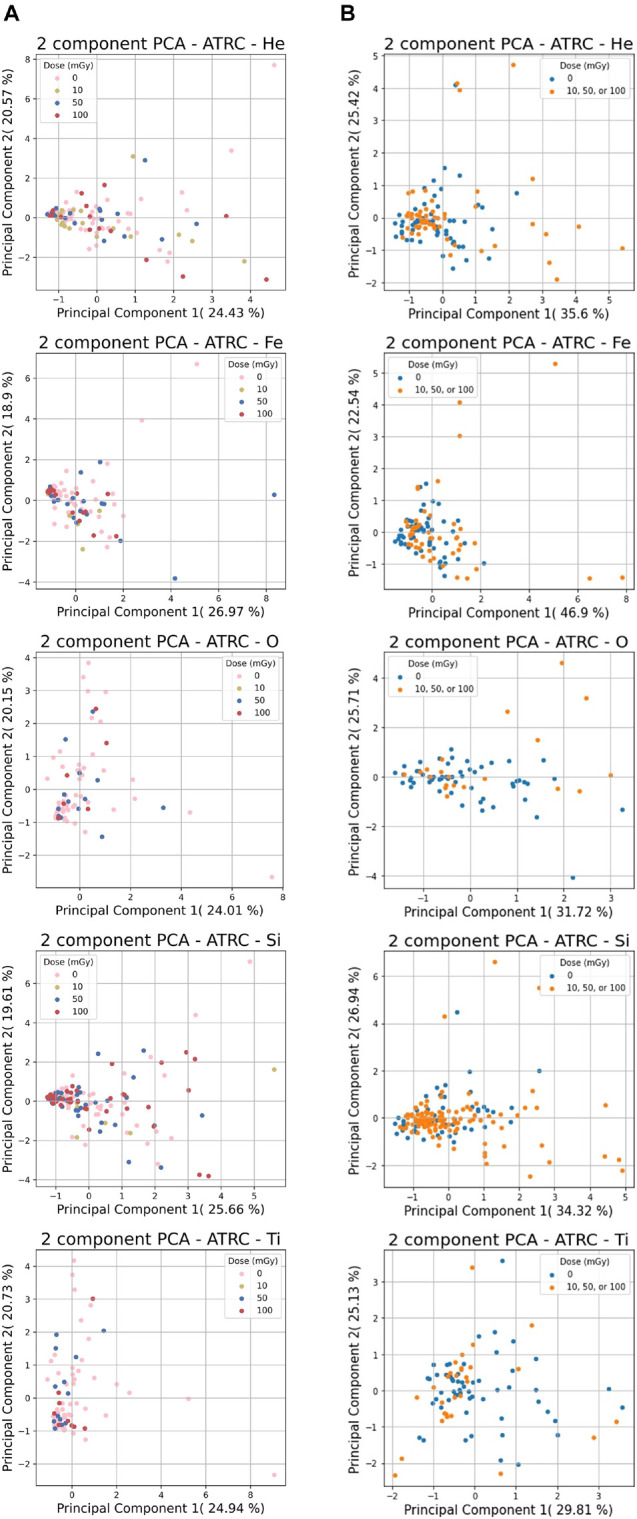
PCA analysis results for the ATRC scores of sham rats and rats irradiated with various ions. **(A)** shows the 7-D non-normalized raw data for 10, 50, or 100 mGy of each ion and **(B)** shows the same ion data with the 4-stage normalization routine with sham samples in blue and samples irradiated with any dose in orange. The percentages next to the PC number are the percentage of overall variability that is explained by said PC. Principal components 1 and 2 are shown since these reflect the highest variation in the data.

Figure 5 shows a 2-D PCA visualization of the He, Fe, O, Si, and Ti ions datasets using the first two principal components. Since each ion-related dataset consists of 7-D or 4-D features, PCA analysis allows data to be visualized in a lower-dimensional feature space. The 1st principal component is the projection of the data onto the direction of the maximum variance, and the 2nd is the projection on the direction with the second-largest variation orthogonal to the first.

Figure 5 illustrates that in most cases, many of the data points, irrespective of class, cluster in a region near the origin in the 2-D feature space plane. Even though many of the sham and the irradiated subjects end up performing similarly, there are also many outliers outside this general congregation that undergo more pronounced decrement, of which the preponderance of these come from the irradiated subset.

### Classifier Performance

**FIGURE 6:**
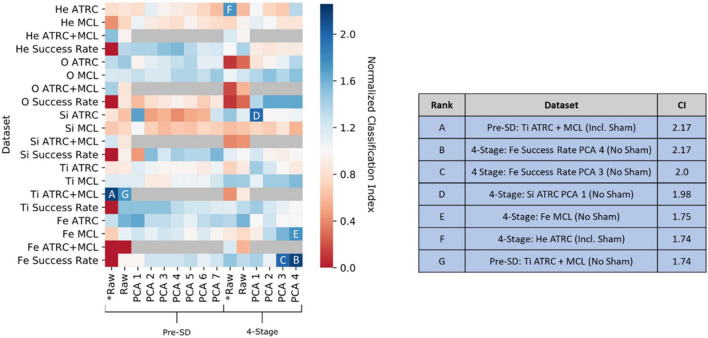
Results of Gaussian naive Bayes using a variety of feature subsets. The *classification index* (CI) is a modified accuracy metric defined as the accuracy of the classifier divided by the probability of achieving this through random chance, that is, the class ratio (the ratio of the number of points of any one class over the number of points from all classes). Dimensionalities in the input vary from 4-D to 14-D (14-D only in the trial case where MCL and ATRC are combined together). * indicates sham data is included. Other cases comprise of classifying the dose as one of 10, 30, 50, 100, and 150 mGy.

Various metrics are aggregated in order to assess a general dose discrimination performance and juxtapose various algorithms and feature sets. Figure 6 and Table 2 present examples of the accuracy results for the SVM algorithm and the GNB analysis, respectively, across different feature set selections and dimensionalities without any class weighting or undersampling procedures performed. The accuracy results seen in this table and figure are better than random chance, but note that class imbalances in the data can often obscure true accuracy performance. Therefore, we evaluate more comprehensively with other metrics as well, such as MCC, to reach a more robust interpretation. This example serves as an illustration of the need for caution when analyzing accuracy results from imbalanced data and an intimation of the need for more robust performance metrics going forward.

**TABLE 2:** Examples of results with the SVM classifier in a 2-D analysis with the Fe ion using the linear kernel without any undersampling or class weighting technique applied.

Test 1	Test 2	Accuracy (%)
SD	CD	62
SD	CDR	65
SD	IDS	71
SD	IDR	56
SD	EDS	59
SD	EDR	62
CDR	IDS	71
CDR	IDR	56
CDR	EDS	50
CDR	EDR	56
IDS	IDR	74
IDS	EDS	71
IDS	EDR	71

*This is an example of predicting the sham vs. any irradiation (10, 30, 50, 100, and 150 mGy) subjects. There are 62 zero dose (sham) and 83 any dose (irradiated rats). This 2-D analysis refers to taking a pair of two ATSET stages as input features at once and predict the zero/any dose in order to investigate if certain subsets of the ATSET stages yield improved classification over others. This creates a total of 21 combinations of stages since there are seven stages of the assay. Accuracies are to be compared to a random chance classification, i.e., the high to low class ratio of 57%.*

We further inspect classifier results by looking at PR, F_1_, and MCC metrics. Table 3 shows model performance metric results from the SVM, GNB, and RF algorithms when applied to the non-normalized ATRC dataset for predicting 0 mGy (sham) vs. any irradiation dose. Overall, the results show consistency in the model performances for particular analyses, with similarly high or low MCC and F_1_ scores. To illustrate, the lowest MCC score overall for all models occur in the He ion prediction (Table 3). Similar tendencies are observed with the other evaluation scores and models. Given the similarity of the model results, for brevity, only the results from the SVM analysis are shown henceforth.

**TABLE 3:** Model performance metrics from the SVM, GNB, and RF algorithms when applied to the non-normalized ATRC dataset for predicting 0 mGy (sham) vs. any amount of irradiation.

Ion	ML model	MCC	F_1_ score
	SVM	0.08	0.38
He	GNB	0.03	0.53
	RF	0.03	0.46
	SVM	0.15	0.34
O	GNB	0.11	0.43
	RF	0.25	0.48
	SVM	0.12	0.65
Si	GNB	0.16	0.70
	RF	0.11	0.81
	SVM	0.08	0.55
Ti	GNB	0.18	0.56
	RF	0.09	0.48
	SVM	0.33	0.52
Fe	GNB	0.24	0.62
	RF	0.22	0.62

*For the SVM model, the regularization term and kernel coefficient set to 1 and.001, respectively, produces the highest MCC results in the most cases overall across all ions and datasets and are hence considered the optimal hyperparameters overall. All models use balanced class weighting. The GNB model uses 1 for the variance smoothing parameter which produces the highest MCC results overall.*

**FIGURE 7:**
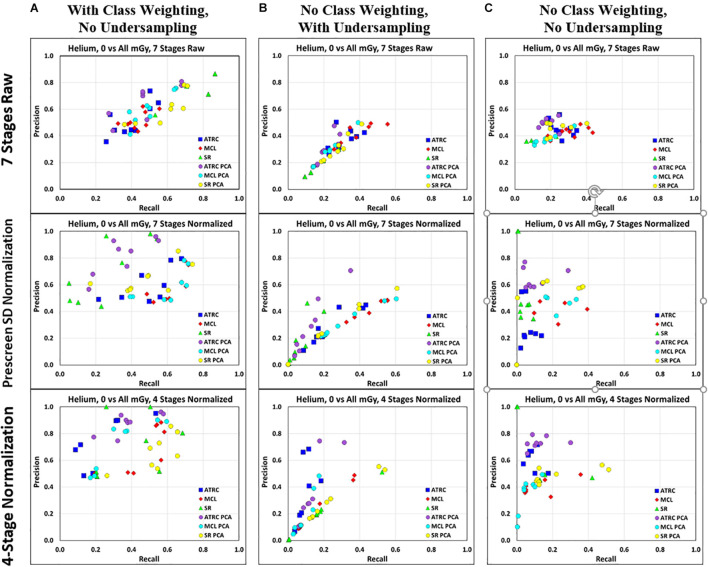
Precision-Recall plots from the SVM classifier created using sham vs. any dose of irradiation of He samples in 3 normalizations (7 stages raw [top row], prescreen SD normalization [middle row], and 4-stage normalization [bottom row]) with class weighting but without under-sampling **(A)**, with undersampling but without class weighting **(B)**, and with neither class weighting nor undersampling **(C)**. Points of the same color and shape represent the same feature scores being utilized but with differing hyperparameter specifications.

Figure 7 shows the PR score results for the SVM algorithm when predicting exposure to He ion between sham (0 mGy) and any dose of irradiation. These PR plots present three different approaches to deal with class imbalances: (1) Class weighting but without undersampling, (2) Undersampling but without class weighting, and (3) Neither class weighting nor undersampling. We compare using class weighting or undersampling and inspecting PR pair plots to evaluate how these remedies affect the classification. PR pair plots and PR curves are a convenient evaluation tool for identifying class imbalance issues in the training stage as one can easily take note of the tradeoffs between precision and recall to discern any apparent aberrations. Obtaining very low precision or recall is often an indicator of class imbalance impacting the model’s training and demonstrates the tradeoff between the two. The results shown in Figure 7C confirm this, given the high precision and low recall scores stemming from class imbalance. The farther up and to the right on the PR pair plots, the better the overall performance of the classifier in that this corresponds to higher precisions and recalls.

One pertinent observation from the analysis is that, when working with class-imbalanced data, evaluation using more class ratio agnostic metrics (e.g., precision, recall, F_1_ scores, MCC, etc.) without using undersampling or class weighting beforehand does not take care of class imbalance effects (even if it did help identify the class imbalance issue more clearly than looking at the accuracy results). That is, just because these more appropriate metrics are utilized does not obviate the need for some other form of mitigation techniques such as undersampling or class weighting. This is sensible because these metrics will not affect the parameter training stage and are merely a retrospective evaluation measure. Another takeaway we note is that class weighting without undersampling yields better results with the SVM classifier across ions and datasets compared to both undersampling alone and neither undersampling nor class weighting (Figure 7). This could be because the samples randomly selected for removal in the undersampling procedure led to missing out on learning specific patterns in the data during training. Without further instruction, we can infer from this that class weighting without undersampling would be our choice in general (when applicable), especially when the data set is particularly large and undersampling routine would be expensive.

We further explore the prediction results by looking at the best performing, i.e., highest MCC, classifiers across all ATSET evaluation scores and hyperparameter combinations (Table 4) to gauge which evaluation scores produce the best results in a particular analysis, and since specific ions and normalizations should have unique model configurations. This subsequent analysis leads to higher MCC and F_1_ score results than those shown in Table 3. After using class weighting, model evaluation metrics reflect performance after class imbalance effects have been mitigated. Overall, the ATRC and Success Rate feature set utilizations lead to the highest MCC, F_1_ score, and accuracy classifier results. MCL produces positive MCC results in only the Ti ion and would hence be considered the least informative metric for purposes of exposure prediction. Moreover, PCA on the corresponding performance scores does lead to improved results in a majority of the cases with the 7-D non-normalized data but in only one case with the 4-stage normalization. We surmise that, consistent with its purposes, PCA aids more in exposure prediction for higher dimensional feature sets than lower ones.

**TABLE 4:** Results of the SVM classifier for predictions of non-irradiated rodents (sham) vs. 100 mGy or any dose irradiated rodents in the best performing, highest MCC, classifier with class weighting applied using ATRC, Success Rate, and MCL.

Ion	Comparison	Dataset normalization	MCC	F_1_ score	Accuracy	ATSET evaluation score
Fe	0 vs. 100 mGy	Non-normalized (raw)	0.23	0.35	78%	Success rate
		4-stage normalization	0.17	0.29	77%	ATRC
	0 vs. Any Irradiation (10, 30, 50, 100, and 150 mGy)	Non-normalized (raw)	0.64	0.79	81%	ATRC
		4-stage normalization	0.61	0.73	78%	ATRC
He	0 vs. 100 mGy	Non-normalized (raw)	0.17	0.37	67%	ATRC
		4-stage normalization	0.25	0.29	79%	ATRC
	0 vs. Any Irradiation (10, 50, and 100 mGy)	Non-normalized (raw)	0.74	0.87	87%	Success Rate
		4-stage normalization	0.60	0.72	78%	ATRC
O	0 vs. 100 mGy	Non-normalized (raw)	0.46	0.51	88%	Success Rate
		4-stage normalization	0.41	0.47	89%	ATRC
	0 vs. Any Irradiation (15, 50, and 100 mGy)	Non-normalized (raw)	0.15	0.34	68%	ATRC
		4-stage normalization	0.22	0.26	74%	ATRC
Si	0 vs. 100 mGy	Non-normalized (raw)	0.08	0.32	59%	ATRC
		4-stage normalization	0.49	0.67	76%	Success rate
	0 vs. Any Irradiation (10, 30, 50, 100, and 150 mGy)	Non-normalized (raw)	0.53	0.81	76%	Success rate
		4-stage normalization	0.47	0.77	72%	Success rate
Ti	0 vs. 100 mGy	Non-normalized (raw)	0.44	0.51	79%	MCL
		4-stage normalization	0.62	0.67	91%	ATRC
	0 vs. Any Irradiation (30, 50, 100, and 150 mGy)	Non-normalized (raw)	0.65	0.79	83%	Success rate
		4-stage normalization	0.77	0.89	88%	Success rate

*The results here are an average across all kernels and includes results with PCA as well. The Comparison column specifies which doses are being examined. Note that not all ions and dose pairs exist in the parent dataset. The Dataset Normalization column specifies which normalization the analysis uses. The Evaluation Score column states which evaluation metric (ATRC, Success Rate, MCL) produces this result.*

## Discussion

In this analysis, we apply three different ML techniques with varying underlying approaches to evaluate consistencies and differences in their exposure prediction capabilities. The results illustrate that the performance varies across algorithm, feature set, and normalization. Overall, the ML classifiers show an ability to successfully discriminate between and predict GCR ion irradiation from the ATSET performance scores better than chance alone (Table 3), suggesting that there are discernable effects between 0 and up to 150 mGy of radiation. All binary predictions between sham and individual doses/any irradiation yield positive correlations between the true class and predicted class (MCC > 0) with the SVM model—a result consistent across all ions. We highlight the ability of each model to discern irradiation from performance scores exceeding random chance rather than hypothesizing whether any of the three particular ML algorithms are preferred over the others in future analyses. We do underscore that the addition of class weighting in the SVM, GNB, and DT models as well as the RF model leads to marked improvement in classifier performance over their respective classic implementations for this imbalanced dataset. As demonstrated by the results of each ML approach to discern radiation exposure from performance scores, the research here demonstrates the feasibility of a data-driven ML approach to analyze and infer the effects of dose and ion on rodent cognitive performance through an inverse methodology to predict exposure using rodents’ performance scores. Working with subject matter experts to develop a cost matrix (Chawla, 2005) that weighs the relative importance of type I and type II classification errors for GCR ion exposure prediction would help elucidate the extent of the significance in this greater-than-chance classifier performance in application. Similarly, a recent parallel effort by our group (Matar et al., 2021) evaluates the susceptibility to cognitive performance impairment in rodents due to space radiation exposure by demonstrating a capability to predict cognitive performance impairment in individual rodents using their respective pre-irradiation performance scores with ML. The findings demonstrate that prescreen performance scores can be used as features with ML to predict ATSET performance impairments as a direct method of predicting impairment.

Performance across normalization routines varies. The analysis shows that ATRC and Success Rate yield the highest performing prediction capabilities overall across all models (Table 4). We find the use of prescreen data using the 4-stage normalization with ATRC augments prediction results over using the ATRC non-normalized data alone in many cases. For instance, 18 out of 18 SVM predictions using varying hyperparameters with the ATRC He ion data produce higher MCC with the prescreen 4-stage normalization applied than with the raw non-normalized data. This would suggest that normalization with prescreen data could be an important step in the prediction of GCR ion exposure from ATRC performance data. The use of the prescreen SD normalization does not yield markedly improved results over the non-normalized and 4-stage normalized datasets and would be excluded from future analyses. MCL only produces the highest MCC classifier in one case (Table 4) and would hence be the least informative metric for exposure prediction purposes.

The findings in this effort are limited to those associated with the data available. To generalize the findings, subsequent research should focus on further validation and inquiry with other datasets, assays, and mixed-field GCR exposure to reach a more robust interpretation of the findings. In particular, the dataset is limited to male subjects whereas studies (Villasana et al., 2010; Krukowski et al., 2018; Parihar et al., 2020) show sex-specific differences in the degree to which space radiation exposure affects cognitive performance. Future work should also include analyzing datasets comprised of both male and female rodents since crewed missions will involve astronauts of both genders. Given these sex-specific effects, sex can be incorporated as an additional feature to inform the models. Not all rodents undergo the same or even any level of cognitive decline. In this analysis, the objective is to predict exposure irrespective of the severity of cognitive decline (if any). We note that at the doses ≤ 150 mGy used in our analysis, subjects have a probability of not being affected by radiation exposure and can be misclassified as sham rats by the ML models. In order to reduce these false negative predictions, one could look at only subjects irradiated above a threshold dose where all animals show a cognitive decline. Such experimental data with higher doses can improve the performance of the ML classifiers but is not relevant to space radiation exposure of astronauts during a 3-year Mars mission, expected to be ≤150 mGy for an individual ion.

A pertinent limitation for this study’s application to space flight decision making is that the ATSET experiment uses single ion exposure and results are not necessarily characteristic of multi-ion GCR exposure which astronauts will encounter in deep space. Combining the effects from single ion irradiation to multi-ion irradiation is still an active and critical area under investigation, which will assuredly provide essential insights for generalizing these results to mixed-field GCR exposure. Another notable limitation in the analysis is the relatively small amount of data (∼700 across all ions) and the inherent noise associated with quantifying rodent behavior and cognitive performance. Both the relatively small size of the dataset and noise can lead to overfitting in the model, which is why cross-validation was employed. Future work would be validation with other datasets using the models and hyperparameters formulated here in order to extract more robust conclusions from the findings. Another future work recommendation would be to assess the predictive capabilities of using subsets of the ATSET by performing a more rigorous 2-D analysis similar to Figure 3 and Table 2 using individual performance score features against other individual performance score features after class weighting and normalization routines are applied. Moreover, methods exist that couple both undersampling and class weighting which could be investigated (Anand et al., 2010). Another assumption to be noted is the use of a uniform prior for the GNB model. This is because the number of samples for each dose is not assumed or expected to be representative of its prior probability. This assumption impacts the model decision-making in that a more representative prior for the exposure samples to be encountered would likely lead to better results. A possible path forward for a spaceflight simulation application would be to have a prior that is integrated by time — i.e., the longer the mission duration, the higher the likelihood that larger radiation doses will be prevalent. This would likely augment the performance of the model by more accurately reflecting the prior probability of exposure.

The translatability from rodents to human models of how to generalize the findings in any murine model or study to humans is often nebulous and undefined given the current state of knowledge (Mak et al., 2014). In terms of future work for this “translation,” we note another line of investigation (Meadows et al., 2008; Lucas et al., 2014) pertaining to radiation exposure’s effect on the change in gene expressions in mice and humans. A machine learning approach adopting a similar methodology to those studies could be leveraged for the purposes of predicting exposure—and potentially other important phenomena—in astronauts subjected to GCR. In this methodology, one would use biomarkers such as gene expressions present in both humans and rodents as a feature set, identify those correlated with radiation exposure, then only use those biomarkers as input to the ML models. This would ideally allow direct prediction and model assessment on human subjects rather than solely on rodents. As a prospective analysis, this current study did not contribute to the experimental design and available features.

One limitation is that the analysis would not necessarily inherit the full benefits of ML in that only the performance scores features are used as inputs in the model. This is sufficient for the purposes described in this study, where the dose and performance are assessed. More generally, training with additional idiosyncratic input features on the rodent individuals, particularly features not directly correlated with their performance, potentially represents new information that captures linkages to existing and unidentified features of the rodent population that are important to assessing performance decrement from GCR ion exposure. This type of idiosyncratic subject-level approach could be valuable considering NASA’s objectives in determining space flight conditions contributing to the overall impact on humans’ cognitive abilities. This relationship, albeit with rodents, has already been observed in literature where a performance decrement transpires in only subsets of the population. For instance, in investigations where the age varies across subjects, performance decrements are often age-dependent (Carrihill-Knoll et al., 2007; Britten et al., 2014; Rabin et al., 2018) and identifying which subsets of the population are more likely to undergo impairment, through ML and its subfields such as anomaly detection, could be a fruitful endeavor to explore in light of the mission objectives. Ascertaining whether performance decrement is unique to a specific subset of the rodent population with particular characteristics would benefit subsequent research by informing that the consequent performance effects of ionizing radiation for astronauts in deep space should be assessed on an individual basis rather than assuming astronauts would be affected uniformly. All of this underscores the ever-accelerating utility of complete and comprehensive subject-level data in rodent and human studies where ML-based approaches like these can be capitalized on and used for prediction.

## Data Availability Statement

The data analyzed in this study is subject to the following licenses/restrictions: US NASA NNX14AE73G, and is being archived in the NASA Life Science Data Archive. Data can be obtained by request at the following link after subsequent request and export control review: https://lsda.jsc.nasa.gov/.

## Author Contributions

MP guided the technical direction of the study, coordinated the modeling implementations, identified standardized approaches for dealing with the imbalanced data, implemented the SVM model, provided critical insights and interpretation of the results throughout, and composed the first draft of the manuscript. MM procured, cleaned, and preprocessed (including PCA) the data in the database, guided the technical direction of the study, identified standardized approaches for dealing with the imbalanced data, provided critical insights and interpretation of the results throughout, and implemented the RF model. SG guided in the direction of the study, managed the logistics of the project and technical task coordination, provided critical insights and interpretation of the results throughout, and served as liaison in communicating our results to the broader NASA and space radiation community. CG implemented the SVM model, provided key visualizations, and contributed to discussions. AS and AI implemented the GNB model. RB conducted the experiments upon which the analyses are based and provided SME interpretations of the experiment and data. RP and BL guided in the direction of the study, guided technical discussions, and provided critical insights and interpretations of the results throughout. JM guided in the direction of the study, guided technical discussions, coordinated logistics, ensured the implementation of the model credibility criteria, and provided critical insights and interpretations of the results throughout. All authors provided continual feedback and review in the manuscript drafting process as well as approved the final manuscript versions.

## Conflict of Interest

During the time of conducting the submitted study, AI and AS were employed by ZIN Technologies, Inc., and RP was employed by the USRA. AI, AS, and RP were employees of contractors funded for this work by the NASA’s Human Research Program. The remaining authors declare that the research was conducted in the absence of any commercial or financial relationships that could be construed as a potential conflict of interest.

## Publisher’s Note

All claims expressed in this article are solely those of the authors and do not necessarily represent those of their affiliated organizations, or those of the publisher, the editors and the reviewers. Any product that may be evaluated in this article, or claim that may be made by its manufacturer, is not guaranteed or endorsed by the publisher.
